# Practical execution of defect preparation prior to surgical cartilage intervention: results from a representative meeting survey among experts

**DOI:** 10.1186/s40064-015-1451-3

**Published:** 2015-11-08

**Authors:** Gian M. Salzmann, Philipp Niemeyer, Stephan Vogt, Peter Kreuz, Markus Arnold, Jürgen Fritz, Ayeesha Mujeeb, Ralf Rosenberger, Matthias Steinwachs, Peter Angele

**Affiliations:** Department of Orthopaedic Surgery, Schulthess Clinic, Lengghalde 2, 8008 Zurich, Switzerland; Department of Orthopedics and Trauma Surgery, Albert-Ludwigs University Medical Center Freiburg, 79106 Freiburg, Germany; Department of Trauma Surgery, University Medical Center Regensburg, Franz Josef Strauß Allee 11, 93042 Regensburg, Germany; Hessinpark Klinik, Augsburg, Germany; Orthopädie, Uniklinik Rostock, Rostock, Germany; Hirslanden Klinik Birshof, Basel, Switzerland; OCC Tübingen, Tübingen, Germany; Department of Biomedical Engineering, College of Engineering, Peking University, 100871 Beijing, China; Universität Innsbruck, Innsbruck, Austria; Hirslanden Klinik Zürich, Zurich, Germany

**Keywords:** Cartilage, Survey, Cartilage repair, Cartilage transplantation, Microfracture

## Abstract

During a specialised orthopedic meeting held on ‘the state of the art in cartilage defect repair’, all previously fully-registered participants were requested to participate in an electronic survey by the use of a moderator-presented “Power Point Presentation-based” 9-item questionnaire. The aim of this survey was to assess indication, approach, and treatment execution of cartilage defect debridement prior to planned microfracture (MFX) or autologous chondrocyte implantation (ACI). All participants completed the questionnaire (n = 146) resulting in a return rate of 100 %. An uncertainty exists as to whether the removal of the calcifying layer prior to cartilage repair must be carried out or not. The same was true for the acceptability of subchondral bleeding prior to microfracturing and its handling prior to autologous chondrocyte implantation. There is a degree of unanimity among experts regarding the management of osteophytes and bone marrow edema. In a homogenous society collective of consultants that frequently deal with cartilage defective pathologies, there still remain a significant heterogeneity in selected topics of defect debridement.

## Background

Cartilage defects are being recognised with increasing frequency (Widuchowski et al. 2007). Apart from the rapid ageing of the population, today’s society is progressively demanding and indulging in more and extreme sporting activity (Filardo et al. 2014). Therefore, cartilage defect treatment is being performed with the same cumulating trend. The spectrum of tools and techniques available on offer for the surgeon has significantly broadened over time (Gobbi et al. 2014; Crawford et al. 2012; Cucchiarini et al. 2014; De Bari and Dell’accio 2008; Khazzam 2013; Abrams et al. 2013; Bhardwaj et al. 2014; Liu et al. 2013). The current evidence is still conflicting when cartilage repair techniques are concerned. However, guidelines are now available as a point of reference (Niemeyer et al. 2013). Yet, such are naturally developed by a local group of specialists and thus have not necessarily to be accepted on an international level. Furthermore, these do include the traditional techniques of microfracture, chondrocyte transplantation and osteochondral transplantation; not considering more novel techniques available in the market. Opposing the emergence of novelty, one is in danger to neglect basic techniques (Gomoll and Minas 2014; Hindle et al. 2014; Pietschmann et al. 2014) of cartilage repair which include patient selection, addressing co-pathologies, rehabilitation and foremost elementary surgical techniques. Such basics have to be considered equally important in order to realise optimal treatment outcome for the benefit of the patient. Additionally, quality evidence is required to accompany operative conversion in the operating room. Prior to most cartilage repair techniques the defect has to be prepared for an optimal consecutive regeneration, independent of technical intention (Gomoll et al. 2011, 2012a, 2012b; Gomoll and Minas 2014; Henn and Gomoll 2011). The majority of these contain statements such as ‘removal of defective cartilage and calcifying layer down to the subchondral bone’, ‘preparing stable surrounding cartilage’, ‘avoid bleeding’ or ‘removal of osteophytes’. Interpretation and potential execution of these descriptions does not have to be consequently uniform. To the best of our knowledge, it has not been analysed before. The aim of this particular presented study was to analyse the exact debridement technique prior to planned MFX or ACI, which are the two most prevalent techniques used clinically at this time.

## Methods

At the end of a committee session during the course of a musculoskeletal meeting (Society for Arthroscopy and Joint Surgery = Gesellschaft für Arthroskopie und Gelenkchirurgie; AGA 2013, Wiesbaden, Germany) on the state of the art in cartilage defect repair all previously fully-registered participants were spontaneously involved in an electronic survey by the use of a ‘Power Point Presentation-based’ 9-item questionnaire. Participants had to electronically identify upon submission, but were further blinded for handling the outcome analysis. The questions were previously designed by all members of the AGA cartilage committee in consensus and presented by an independent moderator to the participants. The participants were capable of answering the posed questions independently from their respective seats by the use of an electronic device. The questions were multiple-choice with only one possible answer to choose. Participants were advised not to discuss their choice of answer during the survey with surrounding neighbours. The informants were asked to reference their opinion on treatment of cartilage defects exclusively at the knee joint (with the exception of item 1, see Table 1). This survey was commenced to explore indication and treatment modalities when cartilage defect preparation is concerned among a homogenous population of highly active experienced and accredited musculoskeletal surgeons from the German-speaking society of Arthroscopy (AGA, http://www.aga-online.de). The AGA is a scientific association of physicians and scientists interested in arthroscopy and associated issues (e.g. cartilage repair). The AGA now has more than 3900 members and is thus Europe’s largest professional society for Arthroscopy in display of a representative population. The spontaneous survey included all participants of the session (n = 146). It was a 9-item Power Point-based electronic questionnaire with one question per page (power point slide) (Table 1). Results were directly presented and discussed with all workshop participants after completion of the survey. Data output/analysis was done descriptively without further statistical analysis.

**Table 1:** Overview of the nine questions that were posed to the audience

Frequency of cartilage surgery per year	None	10	10–50	51–100	>100
Preparation prior to MFX	Never	To calcifying layer	Partially remove calcifying layer	Complete removal of calcifying layer	Into subchondral bone
Preparation prior to ACI	Never	To calcifying layer	Partially remove calcifying layer	Complete removal of calcifying layer	Into subchondral bone
Acceptance of subchondral bleeding prior to MFX	No	Spot bleeding	Spread bleeding	n/a	n/a
Acceptance of subchondral bleeding prior to ACI	No	Spot bleeding	Spread bleeding	n/a	n/a
Management of bleeding prior to ACI	No	Manual compression	Electrocauthery	Fibrin glue	Adrenalin
Treatment of intralesional osteophytes prior to ACI	Topic unknown	Ignore	Removal with curette	Removal with burr	Other technique
How deep to remove intralesional osteophytes	Not	Partially over base of subchondral bone	Complete removal a niveau of subchondral bone	Complete removal under niveau of subchondral bone	n/a
Does defect-associated BME affect cartilage defect treatment	Yes	No	n/a	n/a	n/a

*MFX* microfracture, *ACI* autologous chondrocyte implantation

## Results

All session participants completed the questionnaire (n = 146) resulting in a return rate of 100 %. There were n = 130 consultants and n = 16 residents. A total of 121 were from Germany, 10 from Austria, 8 from Switzerland, 3 from Czeck Republic, 2 from Italy, and 1 each from Poland/Slovakia. From the participants, 12.8 % stated to never carry out cartilage repair, 12.8 % chose to conduct less than 10 interventions/year, 38.5 % between 10 and 50 interventions/year, 25.6 % between 51 and 100 interventions/year, and 10.3 % over 100 inteventions/year, respectively. The answers to the questions listed in the questionnaire (Table 1, methods section) were collected in a chronological order and the data was recorded as follows (Table 2). Defect preparation prior to planned microfracture; never (2.7 %), to calcifying layer (27.0 %), partially removing calcifying layer (8.1 %), complete removal (59.5 %), and into subchondral bone (2.7 %). Defect preparation prior to planned ACI; never (0.0 %), to calcifying layer (23.1 %), partially removing calcifying layer (5.1 %), complete removal (61.5 %), and into subchondral bone (10.3 %). Acceptance of subchondral bleeding during preparation prior to planned microfracture; no (5.6 %), spot (88.9 %), and spread (5.6 %). Acceptance of subchondral bleeding during preparation prior to planned ACI; no (40 %), spot (57.5 %), and spread (2.5 %). Management of subchondral bleeding prior to ACI; no therapy (30.2 %), manual compression (20.9 %), electrocauthery (7.0 %), fibrin glue (20.9 %), and adrenalin (20.9 %). Handling of intralesional osteophytes; not known (0.0 %), ignore (0.0 %), removal with curette (73.8 %), removal with burr (23.8 %), and other technique of removal (2.4 %). The removal of intralesional osteophytes according to depth; not (2.0 %), partially over base of subchondral bone (0.0 %), complete removal a niveau (84.3 %), and complete removal below niveau (13.7 %). The final question, indicating whether bone marrow edema influences cartilage defect treatment 85.7 % respondents selected ‘yes’, whereas 14.3 % answered ‘no’.

**Table 2:** Same table as Table 1 with overview of the nine questions that were posed to the audience

Frequency of cartilage surgery per year	None *12.8 %*	10 *12.8 %*	10–50 *38.5 %*	51–100 *25.6 %*	>100 *10.3 %*
Preparation prior to MFX	Never *2.7 %*	To calcifying layer *27.0 %*	Partially remove calcifying layer *8.1 %*	Complete removal of calcifying layer *59.5 %*	Into subchondral bone *2.7 %*
Preparation prior to ACI	Never *0.0 %*	To calcifying layer *23.1 %*	Partially remove calcifying layer *5.1 %*	Complete removal of calcifying layer *61.5 %*	Into subchondral bone *10.3 %*
Acceptance of subchondral bleeding prior to MFX	No *5.6 %*	Spot bleeding *88.9 %*	Spread bleeding 5.6 %	n/a	n/a
Acceptance of subchondral bleeding prior to ACI	No *40.0 %*	Spot bleeding *57.5 %*	Spread bleeding *2.5 %*	n/a	n/a
Management of bleeding prior to ACI	No *30.2 %*	Manual compression *20.9*	Electrocauthery *7.0 %*	Fibrin glue *20.9 %*	Adrenalin *20.9 %*
Treatment of intralesional osteophytes prior to ACI	Unknown *0.0 %*	Ignore *0.0 %*	Removal with curette *73.8 %*	Removal with burr *23.8 %*	Other *2.4 %*
How deep to remove intralesional osteophytes	Not *2.0 %*	Partially over base of subchondral bone *0.0 %*	Complete removal a niveau of subchondral bone *84.3 %*	Complete removal under niveau of subchondral bone *13.7 %*	n/a
Does defect-associated BME affect cartilage defect treatment	Yes *85.7 %*	No *14.3 %*	n/a	n/a	n/a

The percental answering behaviour of all 146 survey participants is now added to every possible answering possibility

Italics indicate answering percentage

## Discussion

The most important finding of this survey was that until today defect preparation prior to planned cartilage defect surgery is not totally uniform among a collective highly specialised joint surgeons (n = 130 consultants). Apparently, it is not totally clear and there is no consensus on how to remove or treat the calcifying layer before MFX or ACI, with approximately 40 % of the participants selecting the wrong answer (the answer which was not defined as correct by the committee members). A similar answering behaviour was true in terms of acceptability of subchondral bleeding prior to microfracturing and its handling prior to autologous chondrocyte implantation. Yet, there was consistent agreement on the surgical handling of osteophytes and the management of existing bone marrow edema in terms of giving the anticipated reply.

In general, such survey data are truly not representative for worldwide surgical execution, however here we have provided circumstances that propose a highly selected collection of consultants with the desire to offer standard care. More than 35 % of the participants stated to perform cartilage surgery between 51 and100 times/year (without single debridement) and another approximately 40 % between 10 and 50 times/year. Such numbers give insight to the fact that such operations are rather frequent and may propose one quarter of all interventions in selected institutions. Furthermore, concomitant cartilage surgery has to be clearly estimated equally important since anterior cruciate (Unay et al. 2014) or medial patellofemoral (Siebold et al. 2014) ligament ruptures are frequently associated with cartilage defects at typical locations. The surgeon in charge should be expected to be in the position to treat and primarily debride these lesions according to current evidence. If such is lacking, decision making can be dependent on a variety of reasons. Concurrently, one’s surgical approach would be performed i.e. in the way it has been taught. By ways of optimal cartilage defect care chances for the patient are believed to rise to prevent unwanted joint degeneration.

Defect preparation is the initial and equally important step prior to repairing techniques. Frisbie and colleagues have studied the microfracture technique within large, full-thickness medial femoral condyle articular cartilage defects in an equine model in order to mimic lesions often observed in human patients. The horse may come closest to the clinical situation among currently available and accepted animal models, and thus has to be regarded representative (Chu et al. 2010). However, one has to notice that there is always a certain disparity between animal models and a clinical situation. The group (Frisbie et al. 2006) divided horses (n = 12) into two different groups. The first group was microfracture without prior removal of the calcified layer. The second group was with prior removal below the tidemark. The final repair tissue was assessed by the use of arthroscopy, clinical examination, radiographic, and magnetic resonance imaging examinations, biopsy (at 4 months), gross and histopathologic examinations (at 12 months), and finally mRNA as well as immunohistochemical evaluations. When the calcified cartilage layer was removed and the subchondral bone plate was left intact the authors identified an increased overall repair tissue during arthroscopic (4 months) and gross evaluation (12 months). Yet, when the calcified layer was removed an increase in the level of the subchondral bone was still observed. The authors concluded that a removal of the calcified cartilage layer may provide an optimal amount and attachment of the resulting repair tissue. Previous experimental data underline this information where less repair tissue attachment had been seen in a study in which small areas of calcified cartilage were presumably not debrided at the time of defect creation. Also in that same previous study, an increase in tissue filling but no difference in tissue morphology was observed when comparing lesions that were or were not microfractured to the level of the subchondral bone plate (Frisbie et al. 2003). Independently, authors have reported on frequent intralesional osteophyte formation in the aftermath of microfracture application under clinical circumstances (Pestka et al. 2012; Minas et al. 2009), which may be connected to remodelling processes at the subchondral bone. While this statement is not clearly underlined by current literature, it is equally important to retain the subchondral bone after removal of the calcified layer, which has been provided by another study conducted on horses (Hendrix et al. 2010). A recent survey among Canadian orthopedic surgeons revealed comparable, to our survey, interesting information since only 69 % of respondents removed the calcified cartilage layer prior to creating the perforations when performing microfracture surgery (Theodoropoulos et al. 2012). A current study by Mika and co-workers revealed in a controlled laboratory study involving sheep and humans that traditional debridement techniques for ACI using a ring curette do not violate the normal subchondral bone plate in vitro or in vivo.

It has been well established and verified by multiple in vitro and in vivo studies (Forsyth et al. 2012) that joint hematoma following e.g. trauma or among hemophilic subjects has clear detrimental effects, in particular with the articulating cartilage (Sward et al. 2014). With the idea of ACI and its associated technical aspects such as bleeding Sosio and co-workers expanded swine articular chondrocytes and seeded those onto collagen membranes. The membranes were cultured for 3 days in the presence of different concentrations of peripheral blood. All seeded samples showed an increase in weight. Furthermore the authors noted an evident cartilage-like matrix production. A concentration-dependent decrease in the mitochondrial activity related to blood contact was shown at earlier time points of culture. The authors concluded that blood contact of 3 days affected the chondrocytes’ activity. It induced a delay in the maturation of the engineered cartilage constructs (Sosio et al. 2011). In parallel, the topic of intralesional or subchondral bleeding during cartilage defect repair can be found in almost every technical description. The theory behind this is that unwanted blood may interfere with the development of high quality cartilage, which by anatomic definition is without vessels. Since blood within the defect is wanted following MFX and the fraction of stem cells has been reported to be very low one may speculate that elevation of the subchondral plate and intralesional osteophytes may be related to such hematoma. Interestingly, the literary evidence on that topic is very scarce. There is actually no article comparing intra-defect bleeding and consecutive ACI versus no bleeding and consecutive ACI. There is one report comparing in a small animal model two different surgical techniques during articular cartilage defect repair. The purpose of the study was to observe the difference in healing of full-thickness articular cartilage defects treated with burr arthroplasty versus subchondral drilling. Cartilage was shaved off the medial femoral condyles of 39 rabbits without penetrating the subchondral plate. In left knees, two 2.0-mm holes were drilled into the femoral condyle until bleeding was generated. The right knees underwent burr arthroplasty until the authors identified punctate bleeding. The animals were sacrificed at 6, 12, and 24 weeks postoperatively. Joint resurfacing and degenerative alterations were evaluated macroscopically and histologically. Degenerative changes in the cartilage surface were observed within both treatment groups. Rabbits that underwent subchondral drilling had increased fibrocartilaginous healing with a slight increase in degenerative changes across the joint. Burr arthroplasty subjects showed a significant decrease in cartilaginous coverage of the exposed joint surface as well as a progressive increase in direct degenerative changes. Although both techniques have to be declared suboptimal, histological evidence at 6 months does propose that subchondral drilling may result in a better repair than abrasion arthroplasty during the treatment of full-thickness lesions (Menche et al. 1996).

The published nebulosity continues when treatment of intralesional osteophytes is illuminated. Periarticular osteophytes are very well-studied and clearly linked to aging, trauma, mechanical stress, and disease (van der Kraan and van den Berg 2007). Interestingly, elevation of the subchondral bone plate, intra-lesional bony overgrowth (BO) or intralesional osteophytes associated with cartilage repair have never been systematically studied. The true etiology remains unknown. Bony overgrowth rates of incidence linked to cartilage repair have been presented with a large range in variation from 25–70 % for bone marrow stimulation, and from 23–64 % after ACI not necessarily being linked only to violation of the subchondral bone plate. A recent article by Shive reported on much less frequency of BO following MFX surgery and stated that such conditions may not essentially be related to penetration of the underlying bone, but to other factors such as debridement, bleeding, and conditions that existed before the procedure. No relationship to the final clinical outcome was present at 12 months in that study (Shive et al. 2014).

Over 80 % of respondents declared that cartilage-defect associated BME does affect cartilage repair. Until today it is still not clear what a BME does represent (Roemer et al. 2009, 2014; Zanetti et al. 2000) and the evidence for the impact on outcome is conflicting. While Niemeyer (Niemeyer et al. 2010) stated initially that BME at time of ACI is connected to a decreased clinical outcome, the opposite has just lately been reported by Ebert (Ebert et al. 2014). Furthermore, Nemec (Nemec et al. 2009) and Salzmann (Salzmann et al. 2014) have reported that a regional BME is very frequent following cartilage repair (OCT and ACI, respectively), but not connected to clinical or MRI outcome.

In conclusion, we found that there exists a heterogeneous opinion when defect debridement prior to cartilage repair is concerned. The current evidence is in display of a comparable heterogeneity. More research and consequent guidelines are needed in order to harmonise surgical steps during cartilage repair in the future.
